# *Rhizobium* Impacts on Seed Productivity, Quality, and Protection of *Pisum sativum* upon Disease Stress Caused by *Didymella pinodes*: Phenotypic, Proteomic, and Metabolomic Traits

**DOI:** 10.3389/fpls.2017.01961

**Published:** 2017-11-15

**Authors:** Nima Ranjbar Sistani, Hans-Peter Kaul, Getinet Desalegn, Stefanie Wienkoop

**Affiliations:** ^1^Molecular Systems Biology, Department of Ecogenomics and Systems Biology, Faculty of Life Sciences, University of Vienna, Vienna, Austria; ^2^Department of Crop Sciences, University of Natural Resources and Life Sciences, Vienna Vienna, Austria

**Keywords:** pea, seed, *Rhizobium*, *Didymella pinodes*, metabolomics, proteomics, LEA

## Abstract

In field peas, ascochyta blight is one of the most common fungal diseases caused by *Didymella pinodes.* Despite the high diversity of pea cultivars, only little resistance has been developed until to date, still leading to significant losses in grain yield. Rhizobia as plant growth promoting endosymbionts are the main partners for establishment of symbiosis with pea plants. The key role of *Rhizobium* as an effective nitrogen source for legumes seed quality and quantity improvement is in line with sustainable agriculture and food security programs. Besides these growth promoting effects, *Rhizobium* symbiosis has been shown to have a priming impact on the plants immune system that enhances resistance against environmental perturbations. This is the first integrative study that investigates the effect of *Rhizobium leguminosarum* bv. *viceae (Rlv*) on phenotypic seed quality, quantity and fungal disease in pot grown pea (*Pisum sativum*) cultivars with two different resistance levels against *D. pinodes* through metabolomics and proteomics analyses. In addition, the pathogen effects on seed quantity components and quality are assessed at morphological and molecular level. *Rhizobium* inoculation decreased disease severity by significant reduction of seed infection level. *Rhizobium* symbiont enhanced yield through increased seed fresh and dry weights based on better seed filling. *Rhizobium* inoculation also induced changes in seed proteome and metabolome involved in enhanced *P. sativum* resistance level against *D. pinodes*. Besides increased redox and cell wall adjustments light is shed on the role of late embryogenesis abundant proteins and metabolites such as the seed triterpenoid Soyasapogenol. The results of this study open new insights into the significance of symbiotic *Rhizobium* interactions for crop yield, health and seed quality enhancement and reveal new metabolite candidates involved in pathogen resistance.

## Introduction

Legumes are the most important vegetable protein sources in food security programs (Kücük and Kivanc, 2008) as well as European animal feed production (Martin, 2014). They play a key role in the improvement of sustainable agriculture (Kumar et al., 2011). Also, the cultivation of legumes improves soil, decreases the use of nitrogen fertilizers and production costs (Ahmed et al., 2007). Among grain legumes, aboveground biomass of productive pea (*Pisum sativum* L.) holds 300 kg N ha^-1^ while 70% of this amount is in seeds and thus, for this high yielding potential, pea is a key plant in sustainable agriculture (Zajac et al., 2013). The pea key agro-ecological services are linked with its ability to develop symbiotic nitrogen fixation with rhizobia bacteria.

Plant diseases are global threats for limited food supplies and thus implementation of disease management in an effective and sustainable framework is inevitable (Strange and Scott, 2005). Ascochyta blight caused by *Didymella pinodes* is one of the most damaging diseases of pea (*P. sativum*) in major pea planting regions of the world (Setti et al., 2010; Ahmed et al., 2015). This disease reduces seed quality and causes severe yield losses (Garry et al., 1996). Breeding strategies are recommended alternatives to chemical application in disease management of *D. pinodes*. However, low resistance levels in pea cultivars in addition to pathogenic variation in population’s results in ineffective improvement of cultivar resistant (Xue and Warkentin, 2001). Due to the life cycle of disease as primary inoculum causing transmission of infection and in order to protect grain production, a few researches have studied the impact of biological control in disease management of seed infection caused by *D. pinodes* (Moussart et al., 1998).

The endophytic and rhizospheric bacteria with ability to effect on plant growth promotion and reduction of plant stresses are called plant growth promoting rhizobacteria (PGPR) (Orrell and Bennett, 2013). Previous studies have indicated that PGPR increase the resistance level with antagonistic activity of plants against pathogens and herbivores (Orrell and Bennett, 2013).

Apart from nitrogen fixation and plant growth promotion in soybean, *Rhizobium* serves as key antagonist against fungal disease caused by *Fusarium solani*, *Macrophomina phaseolina*, and *Rhizoctonia solani* (Omar and Abd-Alla, 1998; Al-Ani et al., 2012; Das et al., 2017). Seed treatment with *Rhizobium leguminosarum* notably decreases root rot disease caused by *Fusarium solani* f. sp. *phaseoli* of bean and *Pythium* in pea plants (Estevez de Jensen et al., 2002; Huang and Erickson, 2007; Das et al., 2017). Moreover, *Rhizobium* strains are used for biological control of chickpea diseases caused by *Fusarium oxysporum* f. sp. *ciceris*, *Fusarium* spp., *F. solani*, *M. phaseolina*, *Pythium* sp., *Rhizoctonia bataticola*, and *R. solani* (Nautiyal, 1997; Arfaoui et al., 2005; Singh et al., 2010; Shaban and El-Bramawy, 2011; Smitha and Singh, 2014; Das et al., 2017). Thus, *Rhizobium* inoculation may be an efficient, safe and economic alternative to fungicides as bio-control agent in plant disease management (Kumar et al., 2011; Al-Ani et al., 2012). In addition, *Rhizobium* inoculation of fenugreek enhanced the quantity and quality of the seeds (Abdelgani et al., 1999).

The PGPR promote and enhance some growth parameters such as seed germination, seedling vigor, emergence, plant stand, root and shoot growth, total biomass of the plants and seed weight (Kumar et al., 2011). *Rhizobium* as PGPR is a key factor for establishment of symbiosis with legumes. Their role in nitrogen fixation makes them a main component and biological nutrient source in sustainable agriculture. Inoculation of legumes with these bacteria increases biological nitrogen fixation in agriculture, especially in N depleted soils (Neo et al., 2012). Rhizobia are the most notable bacterial symbionts in the rhizoplane, rhizosphere of legumes and surround seeds (Mel’nikova and Omel’chuk, 2009).

Supplying enough high quality seed is crucial in crop establishment and producing high yield as well as food and nutritional security programs across the globe. Some previous studies have shown that *Rhizobium* spp. inoculation of fenugreek and been plants improves seed yield, protein content, and seed quality significantly (Abdelgani et al., 1999; Kücük and Kivanc, 2008). Also, the symbiosis of legumes and *Bradyrhizobium japonicum* increases the biomass of the green parts in inoculated plants at bud stage (Mel’nikova and Omel’chuk, 2009). The plant responses to *Rhizobium* inoculation depends on bacterial strain (s) and plant host varieties (Begum et al., 2001a). There is a knowledge gap in integrative assessment of *Rhizobium* potential as PGPR on all yield components and plant growth parameters in a symbiosis relation with pea plants and against aboveground pathogens at molecular level through seed metabolomics and proteomics analyses. Moreover, little data is known about seed metabolome and proteome in legumes hosting microbial symbionts. Thus, there is a need to understand deeply the effect of *Rhizobium* as belowground microsymbiont on aboveground parts of pea plants and evaluation of its ability to control fungal diseases of plants.

This study evaluates the effect of root nodulating *Rhizobium* as a PGPR microsymbiont on several quantity and quality components including seed metabolomics and proteomics analyses against ascochyta blight disease caused by *D. pinodes* in uninfected (healthy) comparing to infected (diseased) pea plants grown in pots for two cultivars with different levels of resistance.

We focus on the following questions through integrated molecular and phenotypical approaches (a) does *Rhizobium* promote seed yield and quality in two pea cultivars of different susceptibility to *D. pinodes* infection; (b) how does pathogen infection influence seed quantity and quality; (c) does *Rhizobium* inoculation influence pathogen seed infection and how, and (d) are the effects of *Rhizobium* inoculation plant cultivar specific?

## Materials and Methods

### Experimental Design and Treatments

**FIGURE 1:**
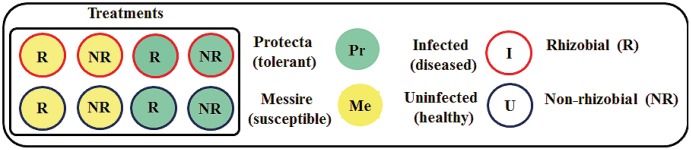
Schematic overview of the experimental setup. Each treatment consisted of three pots. Each pot contained four plants. For morphological experiments *n* = 12 biological replicates per treatment. For molecular experiments *n* = 3 (seeds were pooled from four plants per pot and from three pots per treatment = three biological replicates. Each biological replicate was measured two times = two technical replicates).

To establish the *Rhizobium* – host plant–pathogen interactions, an experimental design was implemented, including three factors (cultivar, microsymbiont, and pathogen) with three pots per treatment and four plants per pot were prepared (**Figure 1**): Messire (Me), susceptible or Protecta (Pr), tolerant); seed treatments as the second [R = rhizobial-inoculated (with *Rlv*) or NR = non-rhizobial-inoculated]; the pathogen *D. pinodes* as the third [U = uninfected (healthy) or I = infected (diseased)].

### Plant Growth Conditions (Pot Experiment)

The *P. sativum* seeds of cultivars Messire and Protecta were received from Rubiales Lab, Cordoba (Spain) and University of Natural Resources and Life Sciences (Austria), respectively. Cultivar Messire has been described as susceptible to *D. pinodes* (Fondevilla et al., 2011), while cultivar Protecta was considered as partially resistant. Seeds of *P. sativum* were surface sterilized in ethanol (95%) for 5 min and then rinsed by sterile water. After that, seeds were immersed in commercial bleach (5%) for 20 min and washed five times with sterile water (Begum et al., 2001a). Seeds were imbibed for 4 h prior to sowing (Begum et al., 2001a). Then, seeds were pre-germinated in trays including sterile mixture of vermiculite and perlite. Then, 3 days old germinated seeds were transplanted into the pots (3 L) with 2 kg pot^-1^ sterile soil (pH 5.6, N 7 mg/L, P 13 mg/L, and K_2_O 120 mg/L) as described in our previous study (Desalegn et al., 2016) and placed under controlled environmental conditions (14-h day/10-h night; 600 μmol m^-2^ s^-1^ light intensity; 22°C/16°C day/night temperature; 60–70% relative humidity) (Ludidi et al., 2007; Larrainzar et al., 2014). All NR plants were fertilized once a week with 200 mL of Broughton and Dilworth nutrient solution (Broughton and Dilworth, 1970; Laguerre et al., 2007) containing 10 mM KNO_3_.

### Plants Harvest and Data Collection

The plants were harvested at seed maturity stage. The vegetative and reproductive growth parameters of plants including pod number (per node and per plant), pod size, pod weight, number of seeds per pod and per plant, seed biomass (DW and FW per plant and per seed and the thousand seed weight) as well as flower number were recorded. Also, seed yield, vigor index and percentage of non-soakers and hydration coefficient were analyzed. Moreover, rhizobial population density and root nodule colonization of pea plants were assessed.

### ***Rhizobium*** Inoculation

Commercial inoculant [JOST GmbH, RADICIN N° 4^1^] of *R. leguminosarum* bv. *viceae* (*Rlv*) was added to the seeds (2 mL/seed) in each planting hole (Naeem et al., 2008; Kumar et al., 2011) and according to prescription of the company. For *Rhizobium* treatments, N-free nutrient solution (KH_2_PO_4_ 68 ppm, K_2_SO_4_ 43.5 ppm, Fe-citrate 2.63 ppm, H_3_BO_3_ 0.12 ppm, MgSO_4_⋅7H_2_O 61.65 ppm, CaCl_2_ 147 ppm, ZnSO_4_⋅7H_2_O 0.14 ppm, CoSO_4_⋅7H_2_O 0.028 ppm, CuSO_4_⋅5H_2_O 0.05 ppm, Na_2_MoO_4_⋅2H_2_O 0.024 ppm and MnSO_4_⋅H_2_O 0.17 ppm) was applied weekly (Desalegn et al., 2016).

### Pathogen Inoculum Preparation and Inoculation

The pathogen isolate (*D. pinodes*) was obtained from the Rubiales Lab, Cordoba (Spain) and inoculum was multiplied according to a modified method of Davidson et al. (2012) on potato dextrose agar (PDA) medium amended with Ampicillin (100 μg/mL) and Chloramphenicol (8 μg/mL) at 22°C and 12 h photoperiod. For preparation of spore suspension, sterile distilled water was added to 7-day-old colonies of *D. pinodes* and then surface of colonies were scraped by sterile needles and resulting suspensions were filtered via sterile cheesecloth (Zimmer and Sabourin, 1986; Carrillo et al., 2013). The concentration of conidia in the filtered suspension was adjusted to 3 × 10^5^ spores/mL (Zimmer and Sabourin, 1986; Carrillo et al., 2013). The leaves of 3 weeks old plants were inoculated in separate place with spore suspension containing Tween-20 (120 μL per 100 mL of suspension) and non-inoculated plants were sprayed with Tween-20 and sterile distilled water (Carrillo et al., 2013). To facilitate the infection, plants were covered by a transparent plastic bag for 48 h and after incubation the plants were uncovered (Carrillo et al., 2013; Okorska et al., 2014). The infected plants were kept separately for 7 days at 21 ± 2°C with a 12 h light photoperiod (Garry et al., 1998) and then placed with uninfected plants under controlled environmental conditions (14-h day/10-h night; 600 μmol m^-2^ s^-1^ light intensity; 22°C/16°C day/night temperature; 60–70% relative humidity) (Ludidi et al., 2007; Larrainzar et al., 2014).

### Evaluation of Seed Yield (Quantity) and Physical Properties

To estimate the seed yield, total weight of all harvested seeds per pot was determined (kg pot^-1^) based on a modified formula of seed yield index (Sajid et al., 2012).

In order to investigate the physical properties of obtained seeds from different treatments, 100 harvested seeds were selected randomly and then immersed in tap water (1:4) for 16 h per each treatment (Abdelgani et al., 1999). The non-soakers and hydration coefficient percentages were obtained to evaluate the number of hard (non-soaked) seeds and their absorption potential, respectively, by the following formulas (Abdelgani et al., 1999):

$$\text{Non-soakers index }(\%)=\frac{\text{Weight of non-soaked seeds}}{\text{Initial weight of seeds}}\times 100$$

$$\text{Hydration coefficient }(\%)=\frac{\text{Weight of soaked seeds}}{\text{Initial weight of seeds}}\times 100$$

Thus, a higher hydration coefficient and lower non-soaker are indicators for better seed quality.

### Seed Vigor Index Assessment

The seed vigor index is an important parameter that includes seed germination and seedling growth properties. The vigor index per treatment was determined by the rolled towel technique as described earlier (Farrag and Moharam, 2012) and calculated by following formula (Farrag and Moharam, 2012):

Vigor index = Seed germination rate×(Shoot length+Root length)

### Isolation of Root Nodulating Rhizobacteria

The nodules were collected carefully from roots at harvesting time (pods fully formed stage BBCH 81-88, 12-14 weeks after transplanting) and washed with tap water. These nodules were sterilized with ethanol (95%) for 5–10 s and washed five times with sterile distilled water (Somasegaran and Hoben, 1985; Datta et al., 2015). Then, they were dipped into 0.5% (v/v) sodium hypochlorite for 1 min and washed seven times with Milli-Q (ultrapure) water (Agrawal and Choure, 2011; Datta et al., 2015). The surface sterilized nodules were squeezed and crushed in 5 mL Milli-Q (ultrapure) water in a tube by sterilized glass rod. The resulting milky suspension was streaked on CR-YMA and YEM Agar as selective *Rhizobium* media (Kneen and LaRue, 1983; Agrawal and Choure, 2011; Neo et al., 2012; Zohra and Mourad, 2016) and incubated at 28 ± 1°C for 48–72 h (Deshwal and Chaubey, 2014; Datta et al., 2015). To enumerate the total number of viable cells, standard plate count method and turbidimetric measurement by spectrophotometry were used (Somasegaran and Hoben, 1985; Pitkäjärvi et al., 2003). Single colonies were picked and restreaked on CR-YMA and YEM Agar plates for preparation of pure cultures (Datta et al., 2015).

### Assessment of Disease Severity

Disease assessment was monitored 10 days after pathogen inoculation and determined every 2 weeks during the growing period. Lesions on leaves and stems were monitored and recorded by USB digital microscope (25×–400×, BMSCI, Japan). Area of lesions was calculated using lesions dimension and disease severity was determined based on area of lesions on leaves (Hwang et al., 2006; Ardakani et al., 2009; Carrillo et al., 2013).

### Seed Infection Assay

To assess the seed infection level (100 seeds per treatment), the potato dextrose agar (PDA) and paper towel techniques were applied (Tivoli et al., 1996; Xue et al., 1996; Gaurilčikienė et al., 2012; Mahmoud et al., 2013).

### Seed Secondary Metabolites and Proteome Analysis

#### Seed Harvesting and Sample Preparation

At harvesting time (seed maturity stage), mature seeds from all plants were collected (about 100 seeds per four plants per biological replicate). Freeze dried seeds in liquid nitrogen were ground to a fine powder according to cold milling technique by using Retsch mixer mill MM 400 (Retsch, Germany).

#### Protein Extraction

For protein extraction, we used the optimized protocol of Wienkoop et al. (2008). Fifty milligrams of seed fine powder per replicate (three biological and two technical replicates per treatment) were homogenized in 1.5 mL of fresh extraction buffer including 50 mM Tris-HCl (pH 7.5), 5 mM EDTA, 0.7 M sucrose, 1% (w/v) PVPP, 1 mM PMSF, 5 mM DTT and Milli-Q (ultrapure) water. Then 1.5 mL Roti^®^-Phenol (Carl Roth GmbH, Karlsruhe, Germany) was added and homogenized again. The homogenates were transferred with shortened tips to 15 mL Falcon tubes and were shaken at 4°C for 30 min. The mixed homogenates were centrifuged at 4000 × *g*, 30 min, 4°C and based on soft slow-down mode at the end of centrifugation. The phenolic upper phase was carefully collected after centrifugation and then was transferred to new 15 mL Falcon tubes and were precipitated over night with ice-cold acetone (five times of the supernatant volume) at -20°C. The precipitated proteins were pelleted by centrifugation at 4000 × *g*, 15 min and 4°C. For air-drying of protein pellets, the Falcon tubes were arranged under the fume hood for 10 min and then the pellets were dissolved in 500 μL urea buffer composed of 50 mM HEPES (pH 7.8) and 8 M urea.

#### Bradford Analysis, Protein Digestion, and Desalting

The protein concentration was measured according to Bradford (1976). For protein digestion, 150 μg of proteins were pre-digested with endoproteinase Lys-C sequencing grade (Roche, Germany) for 5 h at 30°C. Samples were diluted to make a final concentration equal to 2M urea by adding trypsin buffer (10% acetonitrile (ACN), 50 mM ammonium bicarbonate, 2 mM CaCl2). Then, 5 μL of Poroszyme immobilized trypsin beads (Applied Biosystems, Germany) added to samples and incubated at 37°C overnight. For desalting, digests were transferred to C18-SPEC 96-well plates (Agilent Technologies) and desalted according to the manufacturer’s instructions. Desalted peptides were dried by vacuum concentrator and stored at -80°C until MS analysis.

#### Nano ESI LC–MS/MS for Protein Identification

One microgram of desalted and dried peptide samples were dissolved in 2% ACN and 0.1% formic acid (FA). Two technical replicates per sample were injected in random order to an EASY-Spray column, 15 cm × 50 μm ID, PepMap C18, >2 μm particles, 100 Å pore size (PepMap RSLC, Thermo Scientific) and separated during a 180 min gradient with a flow rate of 400 nL/min using an 1D nano LC (UltiMate 3000, Thermo Fisher Scientific) coupled to an Orbitrap Elite Hybrid Ion Trap-Orbitrap Mass Spectrometer (Thermo Fisher Scientific, Bremen, Germany) with full scan range 350–1,800 m/z, enabled dynamic exclusion, exclusion duration 60 s, exclusion list size 500, repeat duration 30 s, repeat count 1, 20 MS2 scans, CID (activation type), enabled charge state rejection with rejected unassigned charge state 1, minimum required signal 1,000 and expiration count 1.

#### Protein Identification and Quantification

The resulting RAW files from MS measurement were uploaded to MaxQuant software package (version 1.5.3.8) for protein quantification and identification with the following group specific parameters default setting: maximum missed cleavages 2, five (as maximum number) of variable modifications (acetylation of the N-term and oxidation of methionine) per peptide, label-free quantification (LFQ) minimum ratio 2, first search peptide tolerance 20 ppm, main search peptide tolerance 4.5 ppm, isotope and centroid match tolerance 2 and 8 ppm, respectively. Also the following global parameters settings: stabilized large LFQ ratio 2, activation of required MS/MS for LFQ, minimum six amino acids as peptide length, revert decoy mode, peptide/spectrum match (PSM) and protein false discovery rate (FDR) 0.01. An assembled protein database (FASTA format) from our previous study (Desalegn et al., 2016) was applied in Andromeda search engine. The mass spectrometry proteomics data as well as the Fasta and mercator files have been deposited in the ProteomeXchange Consortium via the PRIDE (Vizcaíno et al., 2016) partner repository with the dataset identifier PXD006617.

#### Secondary Metabolite Polar Phase Extraction

Metabolites extraction according to De Vos et al. (2007) was optimized. One hundred milligrams of seed fine powder per each treatment with three biological replicates were homogenized in 1 mL of fresh extraction buffer (80% MeOH) by placing in ultrasonic bath at low temperature (ultrasonication on a mixture of water and ice) for 10 min. The homogenates were centrifuged at 21000 × *g*, 10 min and then supernatants were transferred to new 2 mL Eppendorf tubes. The samples were dried by vacuum concentrator. The dried samples were resuspended by adding 50 μL 50% MeOH in 0.1% FA and then centrifuged at 21000 × *g*, 10 min. The supernatants were diluted (1:10) with 0.1% FA and 5% MeOH and 3 μL reserpine 5 mg/L (Sigma-Aldrich) was added to each diluted supernatant as quantitative reference standard. The samples were centrifuged (21000 × *g*, 10 min) again and 20 μL of samples carefully transferred to glass micro-vials with integrated micro-inserts for nanoESI LC–MS/MS.

#### NanoESI LC–MS/MS for Polar Metabolite Analysis

The extracted metabolites (three biological and two technical replicates per treatment) were applied with C18, 2.7 μm (particle size) HPLC column, 15 cm × 100 μm ID (Supelco analytical, Sigma-Aldrich) and 96 min gradient ranging with 400 nL min^-1^ flow rate. The eluent was analyzed by LTQ-Orbitrap XL Hybrid Ion Trap-Orbitrap Mass Spectrometer (Thermo Fisher Scientific, Germany) with full scan range 130–1,800 m/z, enabled dynamic exclusion, exclusion duration 60 s, exclusion list size 500, repeat duration 30 s, repeat count 1, CID activation type and minimum required signal 50,000.

#### Secondary Metabolites Identification and Quantification

##### Untargeted identification approach using MET-COFEA

In a second strategy, MS RAW files were converted to CDF format by Xcalibur (version 2.3.26) (Thermo Fisher Scientific Inc.). The CDF files were loaded into the LC-MS data analysis tool MET-COFEA (metabolite compound-associated feature extraction and annotation algorithms) (Zhang et al., 2014) with the following parameter configurations: intensity cut-off threshold 10,000, ppm threshold 200 for mass tracing, minimum trace length 6, minimum peak width 6, maximum peak width 50, and peak significance threshold 1.5. Then, the output files of MET-COFEA were transferred to MET-XAlign tool (Zhang et al., 2015) for alignment of annotated compounds.

##### Quantitative approach using ProtMAX alignment and manual peak integration

For relative quantitative data matrix alignment MS RAW files were converted to mzXML format by using MassMatrix mass spectrometric data file conversion tool version 3.9^2^. The resulting mzXML files were used to extract the m/z precursor ratio and retention time information by ProtMAX 2012_rev.2.14 (Egelhofer et al., 2013) and also manual peak integration using Xcalibur 2.2 (Thermo Scientific). Further manual spectral analysis was carried out based on precursor mass of identified compounds that showed significant changes between the treatments, their corresponding sum formula and fragmentation patterns (Wang et al., 2016). In addition, specific tandem MS libraries of plant phenolic compounds (Lei et al., 2015), METLIN (The Scripps Research Institute) and HMDB (Wishart et al., 2013) were used for metabolite identification and cross validation with the compound annotations from MET-COFEA.

### Statistics

For quantification of metabolite and protein data, only those compounds that were found in all replicates of at least one treatment (three biological replicates, 1–2 technical) were selected. Missing values were filled by prior distribution using COVAIN Toolbox (Version 2014-Feb-12/MATLAB R2015a) (Sun and Weckwerth, 2012). The significant differences between treatments were determined by ANOVA followed by Tukey HSD test (*p* < 0.05) for phenotypic, proteomic, and metabolomics studies. Corrected *p*-values (*q*-values; Benjamini Hochberg) are additionally provided for proteins. The multifactor (three-way) ANOVA followed with Tukey HSD test (*p* < 0.05) was used to examine the main effects and interaction of biotic stress (with/without *D. pinodes* infection), cultivar type, rhizobial (R) or non-rhizobial (NR) treatments using STATGRAPHICS Centurion XVI.II. For standard error computation in STATGRAPHICS Centurion XVI.II, the pooled standard deviation is divided by square root of observations number (STATGRAPHICS Centurion XVI.II, Statpoint Technologies, Inc., Warrenton, VA, United States). Outliers were removed using 1.5 times the interquartile range. Principle component analysis (PCA) was carried out using COVAIN Toolbox as mentioned above with log_10_ transformed data. Heatmap and cluster analysis was carried out using RStudio (3.3.0). The average intensity of statistically significant proteins among treatments was scaled (z-transformation). Proteins were hierarchically clustered with Euclidian distance and complete linkage method as described previously (Desalegn et al., 2016) and clusters (1–4) were grouped at a height of 1.3.

## Results

### Assessment of Root Nodule Colonization

The population of isolated *Rhizobium* from the nodules of grown pea roots in pots was between 3.8 × 10^8^ and 4.2 × 10^8^ cfu g^-1^ of nodule (Log_10_ transformed population values between 8.58 and 8.62 cfu g^-1^) (Supplementary Table S1). The abundance of isolated *Rhizobium* from nodules was significantly different between the two cultivars such that the population density in cultivar Messire was significantly higher than in nodules of cultivar Protecta (Supplementary Table S1). Non-infected plants revealed higher nodule number than infected plants, however this was not significant (Supplementary Table S1). Nodule weight was not significantly different at any level of treatments (Supplementary Table S1). A decreased abundance of *Rhizobium* density in the nodules was found in infected compared to non-infected treatments of both cultivars (Supplementary Table S1).

### Impact of *R. leguminosarum* bv. *viceae (Rlv)* Inoculation on Yield Components of Healthy and Diseased *P. sativum* Seeds

The seed yield components such as fresh and dry weights per plant and per pod besides TSW were significantly influenced by microsymbiont factor (**Table 1**). In spite of those increased seed weights, the overall seed number per pod and per plant were not significantly affected by *Rhizobium* inoculation (**Table 1**). However, seed number and yield losses upon pathogen infection were significantly reduced in R compared to NR treated cultivar Protecta (Supplementary Table S2).

**Table 1:** Multifactor (three-way) ANOVA including cultivar, microsymbiont, pathogen factors with main and interaction effects on all seed yield components, plant growth parameters, rhizobial root colonization, and nodulation in grown pea plants in pots.

Source of variation	Main effects						Interactions							
		
	Cultivar		Microsymbiont		Pathogen		Cultivar × Microsymbiont		Cultivar × Pathogen		Microsymbiont × Pathogen		Cultivar × Microsymbiont × Pathogen	
							
	A		B		C		A × B		A × C		B × C		A × B × C	
							
	Mean Square	F-Ratio	Mean Square	F-Ratio	Mean Square	F-Ratio	Mean Square	F-Ratio	Mean Square	F-Ratio	Mean Square	F-Ratio	Mean Square	F-Ratio
Seed number (per plant)	12.04	0.09	22.04	0.17	4,565.04	34.89^∗∗∗^	9.38	0.07	145.04	1.11	222.04	1.70	330.04	2.52
Seed number (per pod)	0.17	5.23^∗^	0.08	2.65	0.03	0.89	0.00	0.06	0.00	0.12	0.01	0.41	0.01	0.37
Seed (FW) per seed (g)	0.00	0.82	0.04	30.67^∗^	0.00	1.17	0.00	1.13	0.00	0.27	0.00	0.13	0.00	2.9
Seed (DW) per seed (g)	0.00	7.63^∗∗^	0.01	19.24^∗∗∗^	0.00	0.37	0.00	0.89	0.00	1.47	0.00	2.01	0.00	0.09
Seed (FW) per plant (g)	136.26	8.32^∗^	179.42	10.96^∗∗^	356.85	21.79^∗∗∗^	47.18	2.88	16.98	1.04	9.20	0.56	0.20	0.01
Seed (DW) per plant (g)	9.29	1.56	44.03	7.38^∗^	128.53	21.54^∗∗∗^	27.60	4.63^∗^	0.92	0.15	5.69	0.95	0.22	0.04
TSW-FW (g)	1,045.21	0.82	38,958.70	30.67^∗∗∗^	1,491.32	1.17	1,437.02	1.13	337.98	0.27	164.59	0.13	3,688.74	2.90
TSW-DW (g)	3,024.16	7.63^∗^	7,627.93	19.24^∗∗∗^	145.52	0.37	353.20	0.89	581.52	1.47	794.77	2.01	35.84	0.09
Seed yield (kg)	1.62	8.32^∗^	2.13	10.96^∗∗^	4.24	21.79^∗∗∗^	0.56	2.88	0.20	1.04	0.11	0.56	0.00	0.01
Vigor index	0.56	3.70	0.16	1.07	1.72	11.33^∗∗^	0.01	0.09	0.01	0.04	0.14	0.93	0.00	0.00
Non-soaker (%)	0.19	8.55^∗∗^	0.04	1.64	0.03	1.15	0.00	0.10	0.01	0.39	0.00	0.16	0.00	0.07
Hydration coefficient (%)	4.11	0.23	2.28	0.13	5.63	0.32	5.42	0.30	1.69	0.09	2.29	0.13	1.96	0.11
Flower number	1,828.76	32.09^∗∗∗^	666.76	11.70^∗∗^	1,989.26	34.91^∗∗∗^	110.51	1.94	65.01	1.14	420.84	7.38	231.26	4.06
Pod number per node	0.02	6.37^∗^	0.00	0.13	0.00	0.14	0.00	0.01	0.00	1.54	0.00	0.04	0.00	0.11
Pod number per plant	2.04	0.14	0.04	0.00	287.04	19.63^∗∗∗^	5.04	0.34	18.38	1.26	35.04	2.40	40.04	2.74
Pod size (cm)	1.18	28.24^∗∗∗^	0.42	10.10^∗∗^	0.41	9.76^∗∗^	0.03	0.70	0.00	0.11	0.02	0.58	0.00	0.11
Pod weight (g)	0.17	2.73	0.31	5.07^∗^	1.22	19.75^∗∗∗^	0.00	0.07	0.03	0.51	0.07	1.14	0.07	1.07
*Rhizobium* density from Nodules	0.00	112.50^∗∗∗^			0.00	40.50^∗∗∗^			0.00	0.50				
Nodule number	0.01	5.34^∗^			0.01	2.61			0.00	0.04				
Nodule weight	0.00	2.10			0.01	4.11			0.00	0.26				


*FW, fresh weight; DW, dry weight; TSW, thousand (1000) seed weight. Asterisks illustrate significant effects (Tukey HSD test; ^∗^*p* < 0.05, ^∗∗^*p* < 0.01 and ^∗∗∗^*p* < 0.001) of factors and their interactions on variables.*

Pod size, pod weight, pod number per plant, and flower number were notably influenced by pathogen factor (**Table 1**) such that these were reduced upon infection (Supplementary Table S3). The cultivar factor had significant impact on number of flowers, which were higher in Protecta while pod number per node and pod size was lower in Protecta compared to Messire (**Table 1** and Supplementary Table S3). Furthermore, microsymbiont had significant influence on pod size, pod weight, and flower number, such that R treatment promoted a general increase of these parameters, respectively (**Table 1** and Supplementary Table S3).

Although there was no significant difference for hydration coefficient and non-soaker percentage of seeds between R and NR of healthy and disease plants, R plants showed a lower non-soaker values under healthy (U) and disease (I) conditions (Supplementary Table S4). The results of multifactor analysis additionally revealed that the vigor index and non-soaker percentage were influenced by the pathogen and cultivar factors, respectively (**Table 1**).

### Effect of *Rhizobium* Inoculation on Seed Infection Level

**FIGURE 2:**
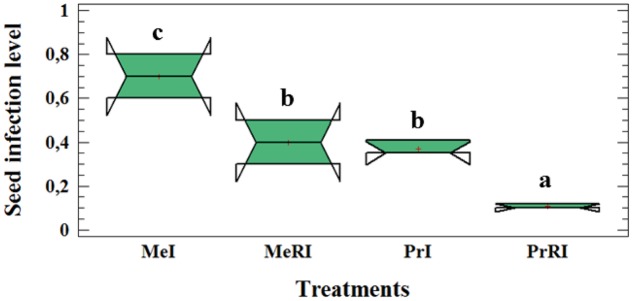
Box-and-Whisker plot (Tukey HSD test, *p* < 0.05, *n* = 12) of significant difference for seed infection severity at cultivar-microsymbiont interaction level of pea plants grown in pots. Me: CV Messire, Pr: CV Protecta, R: rhizobial, NR: non-rhizobial, I: pathogen infected, U: pathogen uninfected. Values with different letter (a, b, c) per treatment are significantly different.

*Rhizobium leguminosarum* bv. *viceae* (*Rlv*) inoculation significantly reduced the seed infection level (∼two fold) compared to non-rhizobial-inoculated (NR) plants (**Figure 2**). We already published results of leaf lesion severity in Turetschek et al. (2017). Here, the same results were determined as for the seed infection level. Our data approved partial resistance of Protecta and susceptibility of Messire against the pathogen. The results of seed infection level assessment of the two cultivars demonstrated that both were infected by *D. pinodes* but the level of seed infection in Protecta was significantly lower than Messire when comparing non-rhizobial-inoculated (NR) or R treatments (**Figure 2**).

The seedling vigor was not significantly affected by rhizobial symbiont but higher vigor index in R plants showed that their seeds were better developed than NR plant seeds (Supplementary Table S4).

Thus, results confirm partial resistance of cultivar Protecta against pathogen infection in comparison with Messire (**Figure 2**).

### Rhizobial Symbiont Impact on the Seed Proteome upon Infected and Uninfected Plants

**FIGURE 3:**
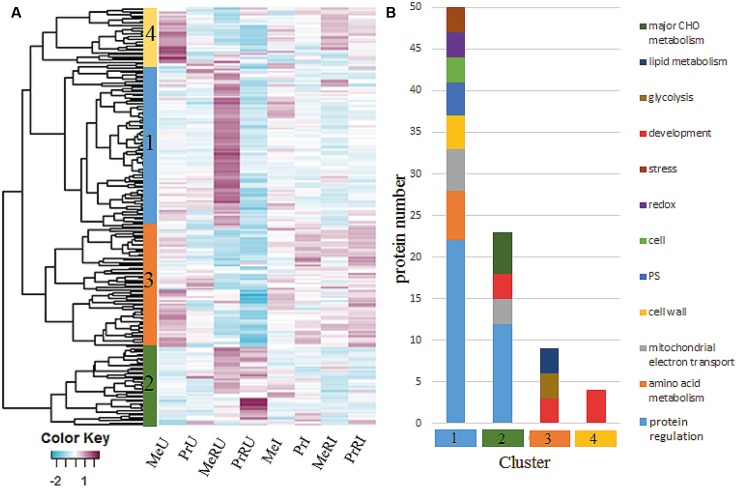
**(A)** Heatmap of 213 protein intensities from seed extractions which illustrated a significant difference among *Rhizobium* treatments of healthy and pathogen infected plants. Each cell indicates the z-transformed average protein intensities (*n* = 3). For cluster analysis, complete linkage and euclidean distance methods were applied; **(B)** clusters from **(A)** were functionally grouped by using functional bins from the MapMan Mercator tool. Categories were plotted when comprising >2 proteins.

In total, 1726 proteins were identified by MaxQuant software package (Version 1.5.3.8). Only proteins without missing LFQ intensity values for at least one treatment were kept for statistical data analysis. All remaining, quantifiable proteins (936) were mapped and functionally classified using the Mercator web pipeline (Lohse et al., 2014). A detailed overview of all identified proteins, assigned functional categories, numbers of proteins and peptides, relative abundance ratios (R to NR treatments) and statistics of pathogen infected and uninfected plants are listed in Supplementary Table S5. About 23% of all quantifiable proteins showed statistically significant differences (ANOVA, Tukey HSD test, *p* < 0.05 and ≥ two fold change) between R and NR comparing both U and I treatments. Abundance changes of those proteins are visualized in **Figure 3A**. Most abundant functional categories for the four clusters are visualized in **Figure 3B**.

Among all identified protein levels of major functional categories (≥3 proteins) TCA, cell wall, plastid, development, and RNA were increased in numbers and fold change in healthy R plants (Supplementary Table S5) independent on cultivar. In addition R treatments of healthy cv. Messire revealed significant induction of proteins involved in protein regulation and amino acid metabolism (**Figure 3**, cluster 1).

Proteins involved in redox regulation were remarkably induced upon pathogen infection in terms of fold-change compared to numbers (Supplementary Table S5), indicative for a hypersensitive response (HR), enhanced in R treated plants. Protein regulation is a major category appearing in both healthy and infected plant processes influenced by R. This category was not different in numbers but showed increased fold changes during infection (Supplementary Table S5). Furthermore, major carbon metabolism followed by DNA and amino acid categories was strongly induced in fold change and numbers of infected R plants (Supplementary Table S5 and **Figure 3**, cluster 2). All these response categories mainly belong to the innate immune system of the plant and are thus typically involved in a basal defense response. However, here several of these proteins are enhanced upon R treatment. Proteins of the development category, especially of the late embryogenesis abundant (LEA) family were most significantly induced mainly in R treated plants upon response to pathogen infection (**Figure 3**, cluster 4), except for cv. Messire, where these proteins showed highest levels in healthy non-*Rhizobium* plants. Proteins involved in development, glycolysis, and lipid metabolism were generally induced upon pathogen attack (**Figure 3**, cluster 3).

### Rhizobial Symbiont Impact on Seed Metabolites upon Infected and Uninfected Plants

Twenty-three metabolites could be identified with high confidence including lipids, flavonoids, anthocyanins, and others (Supplementary Table S6a). In some cases it was, however, not possible to unambiguously identify and distinguish between compounds. Several glycerophospholipids share same masses and formula and could therefore not be discriminated. In these cases, all possible metabolites are listed belonging to the same compound family. Metabolites where evaluated according to significant changes between RU against NRU and RI against NRI (Supplementary Table S6b). Nine metabolites showed significant changes (ANOVA, Tukey HSD test, *p* < 0.05 and fold change ≥ 2) between R and NR treatments being mostly increased either in healthy seeds or during infection (Supplementary Table S6b). The seed terpenoid Pisumoside B was found significantly increase in RU compared to NRU plants. Remarkably, with Soyasapogenol C, ^∗^Api_Dai_Kae_Flavon and 6-Hydroxyapigenin 7-[6″-(3-hydroxy-3-methylglutaryl) glucoside], cultivar Protecta exhibited significantly enhanced changes in RI seeds compared to cv. Messire (Supplementary Table S6b).

### Integrative Rhizobial Symbiont Impact on Seed Proteins, Metabolites, and Phenotypic Parameters of Infected and Uninfected Plants

**FIGURE 4:**
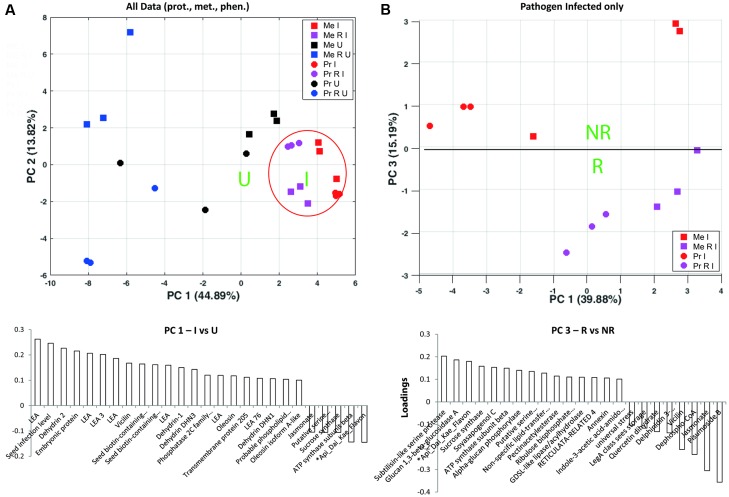
Principle component analysis analyses of **(A)** Seed proteins, metabolites and phenotypic parameters significantly altered between at least two out of all treatments; loadings plot shows compounds with highest loadings of PC1 (loadings > 0.1 and > –0.1). **(B)** Proteins, metabolites, and phenotypic parameters significantly altered between at least two of the pathogen infected treatments; loadings plots shows compounds with highest loadings of PC3 (loadings > 0.1 and > –0.1). Values were log_10_ transformed (Supplementary Table S6); Tukey HSD *p* < 0.05; *n* = 3.

**FIGURE 5:**
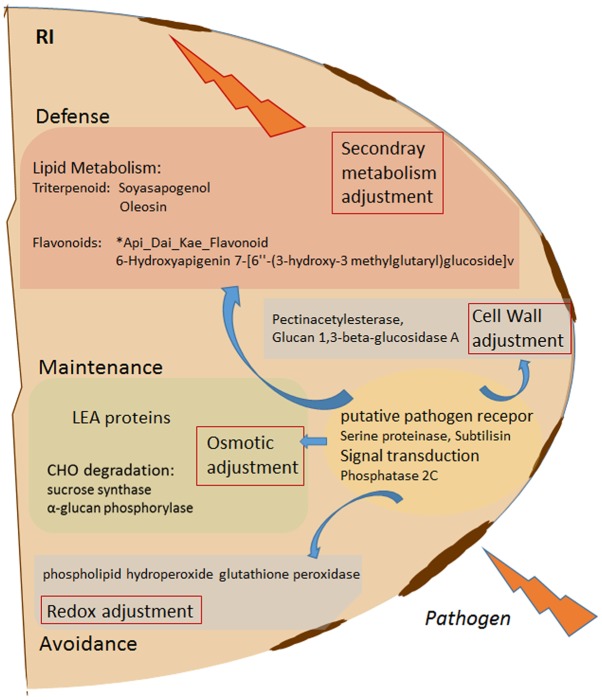
Schematic overview of *Rhizobium* (R) enhanced pathogen (*D. pinodes*) response mechanism in seeds of *Pisum sativum* grown in pots. Selected targets of highest PCA loadings (>0.1 and > –0.1; Supplementary Table S5) of all significant, pathogen responsive and R induced proteins and metabolites (Tukey HSD, *p* < 0.05; *n* = 3; Supplementary Table S5) are functionally illustrated. RI, *Rhizobium* inoculated and pathogen infected; LEA, late embryogenesis abundance; CHO, carbohydrate.

An integrative approach was used to get an overview of those compounds with highest impact on the separation of *D. pinodes* infected to uninfected plants and R treatments. The PCA analyses revealed highest impacts of developmental proteins of the LEA family including LEA and dehydrins accumulating during pathogen infection (**Figure 4A** and Supplementary Table S7a). Notably, LEA abundance levels are much lower in R than in NR treated, healthy (U) plants but most significantly induced during infection. A closer look at the highest impact on pathogen infected treatments revealed a clear separation of R against NR plants by proteins such as sucrose synthase (frv2_111055 also Q9T0M9), serine proteinases such as subtilisin (frv2_80879), Glucan 1, 3-beta-glucosidase A (Q7Z9L3) as well as the metabolites ^∗^Api_Dai_Kae_Flavon and Soyasapogenol, increasing to a higher extend in RI compared to NRI plants (**Figures 4B**, **5** and Supplementary Table S7b). *Rhizobium* specific, especially several storage proteins (vicilin frv2_746012, frv2_130435 and frv2_80171) were found with stronger increase upon infection in cv. Protecta than cv. Messire (Supplementary Table S5) despite the fact that abundance levels were always lower in cv. Protecta and the fact that they showed significantly lower levels in RI compared to NRI in Messire.

## Discussion

Previous studies mostly analyzed the influence of *Didymella pinodes* infection on various non-symbiotic *Pisum sativum* cultivars (Zimmer and Sabourin, 1986; Fondevilla et al., 2005; Carrillo et al., 2013). To the best of our knowledge, this is the most comprehensive study assessing the effects of *Rhizobium* inoculation (R) on the resistance of pot grown *P. sativum* to *D. pinodes*, by integrating seed metabolomics and proteomics with monitoring the various seed infection and yield parameters.

### *R. leguminosarum* bv. *viceae* (*Rlv*) Enhances Seed Yield of Healthy Plants

The promoting impact of *Rhizobium* inoculation on fenugreek seed quality and composition enhancement in addition to induced primary and secondary metabolites of *Glycine max* seeds in plants treated with *Bradyrhizobium japonicum* was described earlier (Abdelgani et al., 1999; Silva et al., 2013). Upon *Rhizobium* inoculation, *P. sativum* seed biomass (DW) was enhanced probably due to an increase in protein and lipid content (Al-Karaki, 2008). Aspartate metabolism has been shown to play a major role during nitrogen nutrition of legume seeds (Atkins et al., 1975). Together with increased amino acid and TCA metabolism our data support that R treatment enhances the level of nitrogen nutrition.

Proteins usually related to the Calvin cycle such as the RuBisCO small subunit were found induced in seeds of R treated plants. Previous studies of legumes already indicate photosynthetic re-assimilation of CO_2_ that substantially contributes to the seeds carbon economy (Schwender et al., 2004; Allen et al., 2009). In contrast, some seed proteins involved in development (mostly LEA proteins; LEA and dehydrin) showed overall lower levels upon R compared to NR treatment in healthy seeds. LEA (incl. dehydrin) proteins usually increase late in plant seed development during desiccation (Olvera-Carrillo et al., 2011). An influence of *Rhizobium* symbiosis on the abundance levels of LEA proteins in seeds has to our knowledge not been reported before.

In general, more proteins were involved in adjustment upon R treatment in cv. Messire (**Figure 3**, clusters 1 and 2) similar to the previous observation for the leaf proteome (Desalegn et al., 2016; Turetschek et al., 2017). The major proteins induced upon R treatment are mainly involved in protein regulation, amino acid metabolism amongst others, which is in line also with our previous studies of *P. sativum* leaves (Desalegn et al., 2016; Turetschek et al., 2017) and of *Medicago* leaves (Staudinger et al., 2016). It indicates that the symbiotic interaction has a direct influence on these processes in leaves and more pronounced for cv. Messire than for cv. Protecta also in seeds. Noticeably, the diterpene glycoside, Pisumoside B, was only found in healthy plants and enhanced through R treatment. Pisumosides have been found in pea seeds earlier, however, their properties in terms of quality remain to be elucidated (Murakami et al., 2001).

In summary, *Rhizobium* as an effective symbiont has an positive and promoting impact on yield components independent of cultivars that has been shown for other bacterial strains before (Begum et al., 2001a; Ahmed et al., 2007; Agrawal and Choure, 2011; Zajac et al., 2013). This study established new insight on proteome level revealing higher levels of primary nutrient metabolism and hydration properties.

### The Pathogen *D. pinodes* Reduces *Rhizobium* Root Nodule Colonization of *R. leguminosarum* bv. *viceae (Rlv)*

We previously analyzed the impact of the pathogen on nodule numbers of the cultivars (Turetschek et al., 2017) without taking *Rhizobium* population into account. Notably, the significantly lower *Rhizobium* population of the nodules of cv. Protecta [Log_10_ transformed population values 8.59 cfu g^-1^] might be balanced by its higher number of nodules. Cultivar specific differences in nodule formation and *Rhizobium* population have also been described before, but for different host plants (Begum et al., 2001b; Bourion et al., 2007; Laguerre et al., 2007).

Interestingly, a stronger impact of nodule number over nodule weight on root biomass enhancement has been described before (Laguerre et al., 2007; Kumar et al., 2011) using different plant host and bacterial strains.

Within the cultivars, reduced abundance of isolated *Rhizobium* from root nodules of infected plants indicate that the fungal pathogen *D. pinodes* had an influence on the efficiency of nodule colonization by *Rlv*, consistent with earlier studies that have found similar results with a different pathogen (*Colletotrichum gloeosporioides*) in *Phaseolus vulgaris* (Ballhorn et al., 2014). A reduced belowground symbiosis was also observed in our previous study that showed even a significant decline in nodule number in cv. Messire (Desalegn et al., 2016). Furthermore, disease reduced growth and growth promoting effectiveness of the symbiotic R compared to NR plants. Thus, *D. pinodes* seemed to hamper symbiotic interaction and thus nutritional exchange and availability also triggered by the reduction of photosynthetic efficiency and thus sugar production induced by the pathogen (Garry et al., 1998). Such reduced R treated activity might downgrade its positive effects on pathogen infection.

### *R. leguminosarum* bv. *viceae (Rlv)* Reduces Disease Severity Independent of Cultivar

It has been shown that enhancement of *Rhizobium* colonization decreases the fungal plant disease severity (Ahmed et al., 2007; Kumar et al., 2011; Desalegn et al., 2016). This bio-control effect of *Rhizobium* was suggested for the disease management of legumes (Al-Ani et al., 2012).

A previous study observed seed-infection that has initially been described as a plant-to-seed transmission of *D. pinodes* (former *Mycosphaerella pinodes*) in field pea (Xue et al., 1997). The level of plant-to-seed infection of ascochyta blight was discussed to be an indication of cultivar specific susceptibility (Marcinkowska et al., 2009). This is in agreement with our actual, where cv. Protecta showed very low levels of seed-infection compared to susceptible cv. Messire independent on R treatment. However, *Rhizobium* inoculated plants showed significantly reduced levels of seed infection caused by *D. pinodes* in both cultivars, which supports the important role of introduced *Rhizobium* as an effective bio-control agent. This is in agreement with our previous findings were proteomics data of cv. Messire revealed a systemic *Rhizobium* -enhanced phytoalexin production in leaves (Desalegn et al., 2016). Nevertheless, it was not clear, whether the reduced disease severity of Messire by *R. leguminosarum* would also reduce yield loss. Although several seed yield parameters such as seed biomass (FW and DW) were enhanced upon *Rhizobium* inoculation of non-infected plants this trend was no longer significant in infected plants. The proteomic data support that seed protective effect against infection through *Rhizobium* increased protein levels (fold change) mainly involved in redox and development (LEA family proteins), major CHO metabolism (starch degradation), mitochondrial electron transport, DNA (protein sequences similar to histones) and cell wall regulation such as subtilisin and a putative pathogen receptor (Figueiredo et al., 2014) (**Figure 5**). *D. pinodes* infection significantly increased levels of thioredoxin (Q9AR82_PEA), L-ascorbate peroxidase (APX1_PEA) and a glutathione peroxidase (similar to U5NF47_CICAR), indicators of oxidative stress induction by the pathogen. Increased levels of endogenous CHO metabolism genes and the hexose to sucrose ratio on treatment of tomato with pathogen *Botrytis cinerea* has been described earlier (Berger et al., 2004). We found sucrose synthase (Q9T0M9_PEA) and an alpha-glucan phosphorylase as the major proteins increased upon pathogen infection in R plants. Other findings confirm the accumulation of proteins related to major CHO metabolism in plant (*P. sativum*)–pathogen interaction (Carrillo et al., 2013; Cerna et al., 2016). These findings support a co-regulation between defense and sink pathways; however, the exact role remains to be elucidated. Interestingly, LEA (and dehydrin) proteins showed highest impacts on the general response to pathogen attack, which has not been shown before. Since several isoforms of these LEA proteins accumulated upon pathogen infection particularly in *Rhizobium* treatments, an important regulatory role of *Rhizobium* on these proteins toward enhanced pathogen resistance can be assumed. Together with the fact that most of them showed lower abundance levels in R compared to NR treatments of healthy plants, this findings suggest that the degree and possibly speed of accumulation is more relevant to increase pathogen resistance than initial level and accumulation itself. Although known to be involved in several stress responses such as drought, LEA proteins were not found related to pathogen defense before. Hence, R plants seemed to influence seed development perhaps by reducing seed dehydration also supported by the enhanced vigor and hydration parameters. LEA proteins have also been reported to be involved in reduction of oxidative stress by scavenging ROS and in protein aggregation and membrane protection (Goyal et al., 2005; Tunnacliffe and Wise, 2007; Tunnacliffe et al., 2010). Disappearance of Pisumoside B upon pathogen infection indicates its involvement in the response process that needs further investigation. Pathogenesis-related (PR) proteins were not found to be involved in seed infection response. Actually, only one PR protein could be identified at all, either because PR proteins are not produced in seeds or because we did not detect them due to other properties such as low abundance or solubility issues. PR proteins are among the most abundantly induced proteins in leaves upon pathogen attack (Van Loon et al., 2006). In our previous study (Desalegn et al., 2016), we found a leaf plasma membrane associated PR protein induced upon pathogen infection.

In our previous study (Desalegn et al., 2016), we found proteins of the pisatin synthesis pathway strongly induced in leaves upon pathogen infection. However, none of these proteins were found induced in this study indicating that pisatin is not synthesized in seeds to protect against *D. pinodes* infection. In fact, we were not able to identify pisatin in the seeds. Presumably, those other protectants play a role here.

Interestingly, our metabolite analysis revealed a seed triterpenoid Soyasapogenol and a seed flavonoid (^∗^Api_Dai_Kae_Flavon) increased significantly and more pronounced in R plants upon pathogen infection. Hence, these secondary metabolites seemed to be involved in defense response against the pathogen attack. However, they were only significantly increased in cv. Protecta. Soyasapogenol was also found in seeds of other pea plants before (Curl et al., 1985). In general, triterpenoids are considered as defensive compounds against pathogens and herbivores (Osbourn, 1996). Its anti-inflammatory, antimicrobial, and cardiovascular-protective activities amongst others, attracts food scientists (Guang et al., 2014). Additionally, several prior studies have described the effective role of non-protein amino acids and anthocyanins in plant defense interactions (Hungria et al., 1991; Bennett and Wallsgrove, 1994; Choung et al., 2001; Troszynska and Ciska, 2002; Dixon and Sumner, 2003; Duenas et al., 2004; Ferraro et al., 2014). Why Soyasapogenol and ^∗^Api_Dai_Kae_Flavon exclusively increased in infected cv. Protecta needs further investigation but may be reason for better protection of that cultivar. Interestingly, quercetin and the phytohormone jasmonate, well known to be involved in plant stress and pathogen defense (Thaler, 2004; Jia et al., 2010), were found increase in R treatments of healthy cv. Messire though not significantly increased upon pathogen infection. Nevertheless, Jasmonate levels were enhanced (though not significant) in R treated, infected cv. Protecta. Interestingly, this correlates with a significant induction of lipoxygenases involved in Jasmonate biosynthesis in this cultivar. Hence, this finding supports jasmonate accumulation, another possible reason for the better performance of cv. Protecta during *D. pinodes* infection.

Taken together, phenotypic, proteomic and metabolomic data provide clear evidence for a *Rhizobium* controlled reduction of seed infection for both cultivars by increased redox, sucrose synthase and LEA proteins. A cultivar specific induction of some proteins and metabolites may explain the improved defense of cv. Protecta such as increase levels of vicilins and Soyasapogenol. This, however, did not clearly influence seed biomass (DW and FW) maintenance upon infection.

It is important to mention, however, that a gap exists between pot and field and field to field studies. The main reason for this is that not all environmental influences relevant for the successful functioning of *Rhizobium* as a bio-control agent are fully understood (Simonsen et al., 2017). Besides climate and plant developmental states, factors such as soil properties, microbial root community-interactions and cultivar specificities may significantly influence on each other and are neither easy to simulate in pots nor transferable from one field to another.

## Conclusion

This research was designed to study the effects of *Rhizobium* symbiont on yield and quality components through seed phenotypic, metabolomic, and proteomic assays in pathogen (*D. pinodes*) infected and non-infected grown *P. sativum* plants in pots.

Together with our previous data (Desalegn et al., 2016), we propose that *Rhizobium* plays an effective role in resistance induction and yield enhancement strategy of *P. sativum* seeds production. Seeds metabolomics and proteomics findings in the current study reveal that seed quality improvement is positively affected upon *Rhizobium* inoculation, even under pathogen infection stress. The impact of the *Rhizobium*-induced protection during defense interactions against pathogen may not only be affected by microsymbiont and genotypic factors but most likely also through other environmental factors such as rainfall, air humidity and temperature. However, these factors need to be studied with more pea cultivars under field experimental conditions in future.

We found that

• *Rhizobium leguminosarum* enhances yield through increased seed fresh and dry weights based on better seed filling. Noticeably the diterpene oligoglycoside, Pisumosides B, was among those compounds increased through *Rhizobium* treatment of healthy pea.
• The pathogen *D. pinodes* reduced root nodule colonization of *R. leguminosarum*. According to our previous study (Desalegn et al., 2016) this indicates that the pathogenic effect is negatively influencing the plant by probably reducing photosynthesis and thus hampers the symbiotic nutrient exchange and its positive impact on seed quantity and quality.
• *Rhizobium leguminosarum* significantly reduces seed infection severity by inducing several proteins and metabolites involved in pathogen response (**Figure 5**). Subtilisin, a putative pathogen receptor, osmotic adjustment via LEA and dehydrin accumulation as well as of proteins involved in carbohydrate degradation, ROS induction, cell wall adjustment, and synthesis of the seed triterpenoid Soyasapogenol as well as the seed flavonoid ^∗^Api_Dai_Kae_Flavon seem key-players during pathogen control. However, effectiveness is cultivar specific.

In order to improve the quantity and quality of food supplies and in line with the global food and nutrition security programs, assessment of the potential of natural and symbiont microorganisms to enhance plant resistance or tolerance capacity against various stresses such as disease as well as promoted plant growth and seed yield in a complex environment, e.g., rhizosphere is considered increasingly relevant in sustainable agriculture. Therefore, the present study not only supports the scattered and limited prior researches, but also provides new evidences and additional understanding with respect to previous studies about the potential effect of *Rhizobium* as key bacterial symbiont to improve and protect yield components and seed quality for sustainable agricultural systems. Further investigations to study the promoting role of *Rhizobium* on growth and yield components in most important legume crops that are considered as main global food sources and on management of economic and epidemic phytopathogens under field conditions are recommended.

## Author Contributions

NRS performed the experiment including phenotypic and molecular analyses. Both NRS and SW analyzed and interpreted the data and wrote the manuscript. H-PK and GD participate in revising the manuscript critically. All authors made substantial contributions to conception and design of the experiment. Authors give final approval of the version to be submitted and any revised version.

## Conflict of Interest Statement

The authors declare that the research was conducted in the absence of any commercial or financial relationships that could be construed as a potential conflict of interest.

## Footnotes

1 http://www.jost-group.com

2 www.massmatrix.org
