# Supplementary material for: Rhizobium Impacts on Seed Productivity, Quality, and Protection of Pisum sativum upon Disease Stress Caused by Didymella pinodes: Phenotypic, Proteomic, and Metabolomic Traits
Source: Front Plant Sci. 2017 Nov 15;8:1961. doi: 10.3389/fpls.2017.01961 (PMC5699443; doi:10.3389/fpls.2017.01961)
Supplement: Supplementary file 1 [file Table_1-4.docx]

Supplementary Material

*Rhizobium* Impacts on Seed Productivity, Quality, and Protection of *Pisum sativum* Upon Disease Stress Caused by *Didymella pinodes*: Phenotypic, Proteomic, and Metabolomic Traits

Nima Ranjbar Sistani, Hans-Peter Kaul, Getinet Desalegn and Stefanie Wienkoop*

*** Correspondence:** Dr. Stefanie Wienkoop: stefanie.wienkoop@univie.ac.at

**
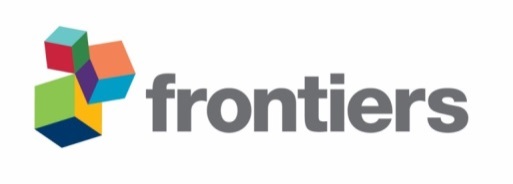
**

**Table S1** The population density of isolated *Rhizobium* from root nodules and nodulation of inoculated pea plants (grown in pots) with *R. leguminosarum* bv*. viceae* (*Rlv*).

| **Treatments** | ***Rhizobium* density from Nodules**  **(log_10_ cfu g^-1^ )^1^** | **Nodule number^2^** | **Nodule weight^2^ (g)** |
| --- | --- | --- | --- |
| **MeRI** | 8.61±0.002 b | 0.66±0.03 a | 1.00±0.02 a |
| **MeRU** | 8.62±0.002 c | 0.70±0.03 a | 1.03±0.02 a |
| **PrRI** | 8.58±0.002 a | 0.72±0.03 a | 1.02±0.02 a |
| **PrRU** | 8.60±0.002 b | 0.77±0.03 a | 1.08±0.02 a |

*Rlv: R. leguminosarum bv. viceae*, Me: CV Messire, I: pathogen infected, U: pathogen uninfected, R: rhizobial, Pr: CV Protecta, NR: non-rhizobial. ^1^ per nodule fresh weight, based on the isolation from all root nodules of 12 plants per treatment, values are log_10_ transformed. ^2^Nodulation was assayed based on the mean of 12 plants per treatment. Values are means ± standard error, (n=12).Values with different letters per each root nodulation index and per each treatment are significantly different (Tukey HSD test, p < 0.05).

**Table S2** Effect of *R. leguminosarum* bv*. viceae* (*Rlv*) on seed growth and yield components in non-infected and infected *Pisum sativum* plants (grown in pots) at treatments level.

| **Treatments** | **Seed number**  **(per plant)** | **Seed number**  **(per pod)** | **Seed (FW)**  **per seed (g)** | **Seed (DW)**  **per seed (g)** | **Seed (FW)**  **per plant (g)** | **Seed (DW)**  **per plant (g)** | **TSW-FW (g)** | **TSW-DW (g)** | **Seed yield (kg)** |
| --- | --- | --- | --- | --- | --- | --- | --- | --- | --- |
| **MeI** | 60.00±6.60 ab | 2.69±0.10 a | 0.36±0.02 a | 0.24±0.01 abc | 21.33±2.34 a | 14.38±1.41 a | 361.64±20.58 a | 243.78±11.49 abc | 2.33±0.25 a |
| **MeRI** | 59.33±6.60 ab | 2.80±0.10 a | 0.48±0.02 c | 0.28±0.01 c | 22.94±2.34 ab | 13.78±1.41 a | 477.25±20.58 c | 278.05±11.49 c | 2.50±0.25 ab |
| **MeU** | 81.33±6.60 b | 2.65±0.10 a | 0.36±0.02 a | 0.22±0.01 ab | 29.67±2.34 abc | 18.23±1.41 ab | 357.92±20.58 a | 219.95±11.49 ab | 3.24±0.25 abc |
| **MeRU** | 83.33±6.60 b | 2.75±0.10 a | 0.43±0.02 abc | 0.27±0.01 bc | 33.39±2.34 bc | 19.96±1.41 ab | 434.42±20.58 abc | 272.34±11.49 bc | 3.64±0.25 bc |
| **PrI** | 45.00±6.60 a | 2.49±0.10 a | 0.41±0.02 abc | 0.22±0.01 ab | 25.16±2.34 ab | 13.68±1.41 a | 407.60±20.58 abc | 221.60±11.49 ab | 2.74±0.25 ab |
| **PrRI** | 61.67±6.60 ab | 2.72±0.10 a | 0.44±0.02 abc | 0.24±0.01 abc | 32.01±2.34 abc | 17.75±1.41 ab | 442.67±20.58 abc | 235.63±11.49 abc | 3.49±0.25 abc |
| **PrU** | 91.00±6.60 b | 2.49±0.10 a | 0.37±0.02 ab | 0.21±0.01 a | 29.77±2.34 abc | 17.13±1.41 ab | 369.31±20.58 ab | 212.57±11.49 a | 3.25±0.25 abc |
| **PrRU** | 80.67±6.60 b | 2.53±0.10 a | 0.46±0.02 bc | 0.25±0.01 abc | 39.46±2.34 c | 22.77±1.41 b | 464.45±20.58 bc | 254.51±11.49 abc | 4.30±0.25 c |

FW: fresh weight, DW: dry weight, TSW: thousand (1000) seed weight, Me: CV Messire, I: pathogen infected, U: pathogen uninfected, R: rhizobial, Pr: CV Protecta, NR: non-rhizobial. Seed growth and yield parameters were assayed based on the mean of 12 plants per treatment (n=12). Values with different letter per treatment and parameter are significantly different (Tukey HSD test, p < 0.05).

**Table S3** Impact of *R. leguminosarum* bv*. viceae* (*Rlv*) on plant growth parameters in non-infected and infected grown plants in pots.

| **Treatments** | **Flower number** | **Pod number**  **per node** | **Pod number**  **per plant** | **Pod size (cm)** | **Pod weight (g)** |
| --- | --- | --- | --- | --- | --- |
| **MeI** | 38.00±4.36 ab | 0.27±0.03 a | 24.00±2.21 ab | 5.45±0.12 abc | 2.23±0.14 ab |
| **MeRI** | 29.67±4.36 a | 0.29±0.03 a | 23.00±2.21 ab | 5.75±0.12 bc | 2.44±0.14 abc |
| **MeU** | 38.33±4.36 ab | 0.25±0.03 a | 29.00±2.21 ab | 5.65±0.12 abc | 2.61±0.14 abc |
| **MeRU** | 59.17±4.36 bcd | 0.24±0.03 a | 28.33±2.21 ab | 6.02±0.12 c | 2.81±0.14 abc |
| **PrI** | 41.67±4.36 abc | 0.20±0.03 a | 19.33±2.21 a | 5.08±0.12 a | 2.20±0.14 a |
| **PrRI** | 54.33±4.36 bc | 0.20±0.03 a | 25.33±2.21 ab | 5.18±0.12 ab | 2.66±0.14 abc |
| **PrU** | 61.00±4.36 cd | 0.21±0.03 a | 33.00±2.21 b | 5.27±0.12 ab | 2.93±0.14 bc |
| **PrRU** | 78.00±4.36 d | 0.22±0.03 a | 29.00±2.21 ab | 5.56±0.12 abc | 2.97±0.14 c |

Me: CV Messire, I: pathogen infected, U: pathogen uninfected (healthy), R: rhizobial, NR: non-rhizobial, Pr: CV Protecta. Plant growth parameters were assayed based on the mean of 12 plants per treatment. Values are means ± standard error, (n=12). Values with different letters per each growth parameter and per each treatment are significantly different (Tukey HSD test, p < 0.05).

**Table S4** Influence of *R. leguminosarum* bv*. viceae* (*Rlv*) on vigor index and physical properties of seed in non-infected and infected grown plants in pots.

| **Treatments** | **Vigor index** | **Non-soaker (%)** | **Hydration coefficient (%)** |
| --- | --- | --- | --- |
| **MeI** | 0.67±0.22 a | 1.42±0.09 a | 8.36±2.44 a |
| **MeRI** | 1.02±0.22 a | 1.32±0.09 a | 8.74±2.44 a |
| **MeU** | 1.38±0.22 a | 1.28±0.09 a | 6.73±2.44 a |
| **MeRU** | 1.44±0.22 a | 1.26±0.09 a | 9.49±2.44 a |
| **PrI** | 1.04±0.22 a | 1.21±0.09 a | 10.10±2.44 a |
| **PrRI** | 1.32±0.22 a | 1.10±0.09 a | 9.72±2.44 a |
| **PrU** | 1.71±0.22 a | 1.17±0.09 a | 8.55±2.44 a |
| **PrRU** | 1.66±0.22 a | 1.08±0.09 a | 8.27±2.44 a |

Me: CV Messire, I: pathogen infected, U: pathogen uninfected, R: rhizobial, Pr: CV Protecta, NR: non-rhizobial. Physical properties indexes were based on the mean of 12 plants per treatment. Values are means ± standard error, (n=12).Values with different letter per each index and per each treatment are significantly different (Tukey HSD test, p < 0.05).
